# Surgical rescues for critical hemopericardium complicated by acute type A aortic dissection: Emergent subxiphoid pericardiotomy or cardiopulmonary bypass first?

**DOI:** 10.1371/journal.pone.0229648

**Published:** 2020-03-02

**Authors:** Chun-Yu Lin, Meng-Yu Wu, Chi-Nan Tseng, Yu-Sheng Chang, Yuan-Chang Liu, Cheng-Hui Lu, Feng-Chun Tsai

**Affiliations:** 1 Department of Medicine, College of Medicine, Chang Gung University, Taoyuan, Taiwan; 2 Department of Cardiothoracic and Vascular Surgery, Chang Gung Memorial Hospital, Linkou Medical Center, Taoyuan, Taiwan; 3 Department of Medical Imaging and Intervention, Chang Gung Memorial Hospital, Linkou Medical Center, Taoyuan, Taiwan; 4 Department of Cardiology, Chang Gung Memorial Hospital, Linkou Medical Center, Taoyuan, Taiwan; IRCCS Policlinico S.Donato, ITALY

## Abstract

**Background:**

Hemopericardium is a common and hazardous complication of acute type A aortic dissection (ATAAD). This retrospective study aimed to clarify the short-term and mid-term outcomes in patients who underwent surgical rescues for hemopericardium complicated by ATAAD.

**Methods:**

Between January 2007 and March 2019, 586 consecutive patients underwent ATAAD repair at our institution. According to preoperative computed tomography, hemopericardium was found in 191 patients (32.6%), 150 were stabilized with medical treatment, and 41 underwent surgical rescues for critical hemodynamics. The 41 patients were classified into groups according to their rescue procedures: emergent subxiphoid pericardiotomy (E-SXP group, n = 26, 63.4%) or emergent cardiopulmonary bypass (E-CPB group, n = 15, 36.6%). Clinical features, surgical information, postoperative complications, and 3-year survival were analyzed and compared.

**Results:**

Demographics, comorbidities and aortic repair procedures were generally homogenous between the two groups, except for sex. The average systolic blood pressure was 62.4 ± 13.3 mmHg and 67.1 ± 13.1 mmHg in the E-SXP and E-CPB groups, respectively. A total of 29.3% of patients underwent cardiopulmonary resuscitation (CPR) before surgical rescues. The in-hospital mortality was similar (30.8% versus 33.3%, *P* = 0.865) in the two groups. Multivariate analysis revealed that preoperative CPR was an in-hospital predictor of mortality. For patients who survived to discharge, 3-year cumulative survival rates were 87.8% ± 8.1% and 60.0% ± 19.7% in the E-SXP and E-CPB groups, respectively (*P* = 0.170).

**Conclusions:**

Patients who underwent surgical rescues for ATAAD-complicated hemopericardium are at a high risk of in-hospital mortality. The two rescue procedures revealed similar short-term and mid-term outcomes.

## Introduction

Acute type A aortic dissection (ATAAD) is a cardiovascular emergency associated with high morbidity and mortality rates. Despite advancements in diagnostic tools, management algorithms, and surgical techniques in recent decades, ATAAD remains challenging for cardiothoracic surgeons. Hemorrhagic leakage from the dissected ascending aorta (AsAo) can accumulate in the pericardial space and lead to cardiac tamponade, which is the most common cause of death among ATAAD patients, according to the International Registry of Acute Aortic Dissection [1]. The reported incidence of hemopericardium ranges from 24% to 66% [2–4]. Once patients present with hemopericardium and stabilization with medical treatment, such as intravenous fluid resuscitation and inotropic agents, fails, a surgical rescue procedure should be promptly implemented to reverse the severely compromised hemodynamics. Otherwise, these patients may not survive to undergo the aortic repair procedures. Emergent subxiphoid pericardiotomy (E-SXP), which decompresses the pericardial cavity and emergent cardiopulmonary bypass (E-CPB), which temporarily supports systemic perfusion are both used as surgical rescue procedures for this critical condition in ATAAD patients. This study aimed to compare the early-term and mid-term outcomes of E-SXP with those of E-CPB, based on a retrospective analysis of the experiences of an individual center.

## Material and methods

### Patient enrolment and preoperative management

The present study was approved by the Chang-Gung Medical Foundation Institutional Ethics Committee (No.201901077B0). The need for informed consent was waived due to the retrospective nature of the study, and all patients’ data were anonymized before being accessed by researchers. Overall, 586 consecutive adult patients underwent emergency ATAAD repair at our institution between January 2007 and March 2019. All patients were diagnosed via helical computed tomography to confirm ATAAD in the emergency department (ED). Extent of aortic dissection, thickness of hemopericardium, and presence of organ malperfusion were analyzed by experienced radiologists. Once the diagnosis of ATAAD was confirmed, patients were emergently transferred to the operating room (OR) within 30 min. If patients presented with hypertension or tachycardia in the OR, their hemodynamics were stabilized with intravenous beta-blockers to maintain systolic blood pressure (SBP) <120 mmHg and heart rate <60 bpm, in accordance with the 2010 American College of Cardiology/American Heart Association guidelines for thoracic aortic disease [5]. When patients presented with shock, medical resuscitation was applied first, including intravenous fluid supplementation and inotropic infusion, such as Dobutamine and Epinephrine to maintain an SBP of >80 mmHg and a heart rate of >50 bpm. Transesophageal echocardiography (TEE) was performed simultaneously by specialized cardiovascular anesthesiologists to quantitatively assess cardiac function, amount of pericardial effusion and the severity of cardiac tamponade. If shock status persisted after medical resuscitation, a surgical rescue procedure was performed.

**Fig 1:**
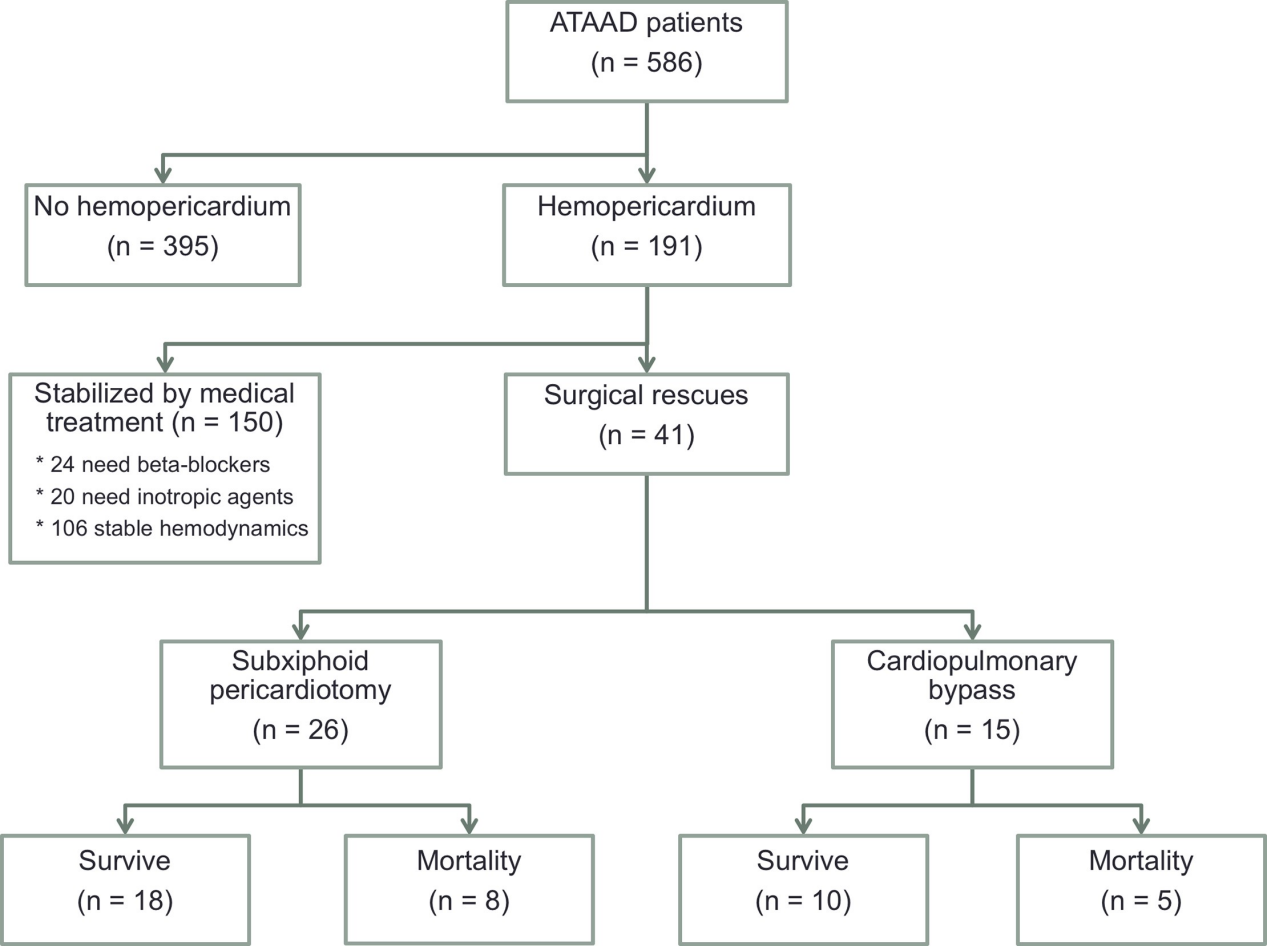
Enrolment, allocation and analysis of acute type A aortic dissection (ATAAD) patients from January 2007 to March 2019.

As illustrated in Fig 1, 395 patients without hemopericardium and 150 stabilized with medical treatment were excluded, and a total of 41 patients underwent emergent surgical rescues for critical cardiac tamponade induced by ATAAD-complicated hemopericardium. The included patients were classified according to their rescue procedures: E-SXP group (n = 26, 63.4%) and E-CPB group (n = 15, 36.6%).

### ATAAD repair and surgical rescue procedures

The aortic repair procedures are detailed in previous studies reported from this institute [6,7]. For patients with stable hemodynamics under medical treatment, the right axillary artery and/or femoral artery were exposed and cannulated with an 8-mm ring-reinforced polytetrafluoroethylene graft. Following sternotomy, the right atrium was cannulated and cardiopulmonary bypass (CPB) with deep hypothermia was initiated. In general, the dissected aorta was replaced with a Dacron prosthetic graft according to the location of the entry tear and preoperative presentation. Concomitant aortic root replacement and frozen elephant trunk procedure were performed in patients with severe aortic regurgitation and descending aortic dissection with malperfusion, respectively.

E-SXP was performed after quick skin sterilization at the lower edge of the sternum. A midline incision was made from the xiphisternal junction to 5–10 cm below the tip of the xiphoid, and the linea alba was divided. A plane was developed behind the xiphoid cartilage, and the xiphoid cartilage was then lifted anteriorly with a retractor. For obese patients, the xiphoid cartilage can be fractured or cut with scissors, and the diaphragm can be depressed with a gauze sponge to improve the exposure of the retrosternal area. Fat was bluntly dissected away until the pericardium appeared as a fibrous membrane [8,9]. With effective visualization of the pericardium, it was penetrated with fine-tipped hemostatic forceps. By spreading the tips of the hemostatic forceps, a 2–4 cm^2^ pericardiotomy was created. After evacuation of the hemopericardium and stabilization of hemodynamics, the xiphoid region was packed with gauze to prevent persistent bleeding from the pericardial cavity, and further aortic repair procedures were implemented immediately. The E-SXP procedure was usually completed in 3 min.

E-CPB was routinely cannulated at the ipsilateral femoral artery and femoral vein. Using a cut-down approach, two wire-guided vascular cannulae (DLP Medtronic, Minneapolis, MN, USA; inflow: 19–23 French, outflow: 17–21 French) were used to establish the femoral-femoral partial cardiopulmonary bypass. Cannulae locations were confirmed by TEE during the cannulation process to prevent great vessel injury. The pump flow was kept at maximum to achieve a mean arterial pressure between 60–80 mmHg. After hemodynamics were stabilized, further aortic repair was implemented. Additional right axillary arterial cannulation was performed and connected with the femoral arterial cannula with a Y-shape circuit when an antegrade cerebral perfusion (ACP) strategy was applied. During circulatory arrest, the femoral arterial flow was temporarily suspended and selective ACP through the right axillary artery was used. The right radial arterial pressure, nasal-pharyngeal temperature, and back flow of left common carotid artery were routinely tested and monitored. The E-CPB procedure was usually completed in 10 min.

### Statistical analyses

We performed statistical analyses with SPSS for Windows (Version 21, IBM, Inc., NY, USA) and set the statistical significance at *P*<0.05 for all analyses. For univariate comparisons, we used the independent T–test or Mann–Whitney U test for numerical variables, and the Chi–square or Fisher’s exact test for categorical variables. We presented the outcomes of univariate comparisons as mean ± standard deviation for numerical variables, and numbers/percentages for categorical variables. We used the multivariate logistic regression method to identify the independent predictors of in-hospital mortality among the collected variables. Finally, we used a Kaplan–Meier survival analysis to estimate the survival outcomes in this patient cohort and the log-rank test to compare the survival data between groups.

## Results

### Patient demographics

**Fig 2:**
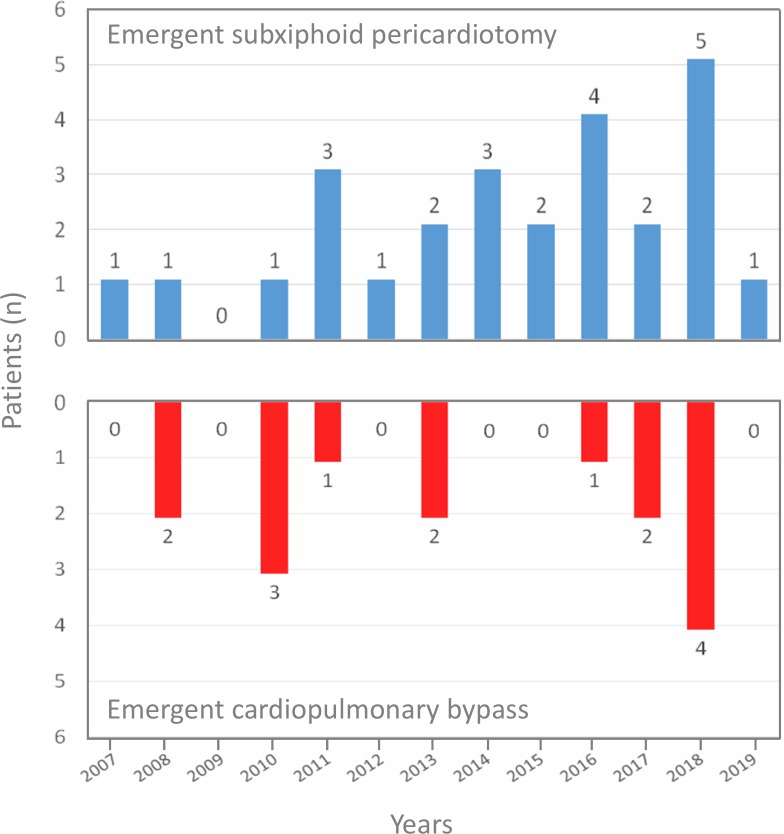
Distribution of rescue procedures during the study period.

As illustrated in Fig 2, both the E-SXP and E-CPB procedures generally revealed an even distribution during the study period. The clinical demographics, comorbidities, preoperative conditions, and clinical presentations for the E-SXP and E-CPB group are illustrated in Table 1. In the E-CPB group, a higher prevalence of female patients was found compared to that in the E-SXP group (34.6% versus 66.7%, *P* = 0.047). The average SBP was 62.4 ± 13.3 mmHg and 67.1 ± 13.1 mmHg in the E-SXP and E-CPB groups, respectively. A total of 29.3% of patients underwent cardiopulmonary resuscitation (CPR) before surgical rescues were performed. Seven patients underwent intubation in the ED, including 1 with unclear consciousness, 2 with inotropic medications for hemodynamic instability, 1 with severe dyspnea, and 3 underwent E-SXP in the ED. The average thickness of the hemopericardium was 15.7 ± 7.9 mm and 12.9 ± 7.4 mm in the E-SXP and E-CPB groups, respectively. Overall, 19.5% of the patients were diagnosed with intramural hematoma and 39.0% were classified as DeBakey type II aortic dissection. Except for hemopericardium, intractable pain was the most common clinical presentation in both groups, followed by aortic regurgitation with heart failure symptoms. No disparity in clinical presentation was found between the two groups.

**Table 1:** Preoperative characteristics according to patient group.

Parameters	Overall	E-SXP	E-CPB	*P* value
	n = 41	n = 26	n = 15	
Clinical demographics				
Sex (female, n, %)	19, 46.3	9, 34.6	10, 66.7	0.047
Age (years)	67.1 ± 14.4	68.7 ± 14.7	64.2 ± 13.9	0.341
Body mass index (kg/m^2^)	25.0 ± 3.8	24.9 ± 3.7	25.2 ± 4.2	0.846
Hypertension (n, %)	31, 75.6	20, 76.9	11, 73.3	0.797
Diabetes mellitus (n, %)	5, 12.2	2, 7.7	3, 20.0	0.246
Creatinine (mg/dl)	1.6 ± 0.8	1.6 ± 0.8	1.4 ± 0.9	0.414
eGFR (ml/min/1.73 m_2_)	52.2 ± 22.5	50.0 ± 19.9	56.2 ± 26.8	0.403
ESRD (n, %)	0	0	0	0.999
Preoperative condition				
SBP (mmHg)	64.1 ± 13.3	62.4 ± 13.3	67.1 ± 13.1	0.286
SBP <60 mmHg (n, %)	17, 41.5	12, 46.2	5, 33.3	0.422
CPR (n, %)	12, 29.3	8, 30.8	4, 26.7	0.781
Ventilator support in ED (n, %)	7, 17.1	3, 11.5	4, 26.7	0.215
Repeat operation (n, %)	1, 2.4	0, 0	1, 6.7	0.183
Time from ED to OR (hr)	4.7 ± 2.4	4.9 ± 2.6	4.4 ± 1.9	0.483
Time from ED to rescue procedure (hr)	5.1 ± 2.5	5.1 ± 2.8	4.9 ± 2.0	0.811
Clinical presentation				
Intractable pain (n, %)	28, 68.3	19, 73.1	9, 60.0	0.386
AR with heart failure (n, %)	7, 17.1	3, 11.5	4, 26.7	0.215
Hemopericardium (n, %)	41, 100	26, 100	15, 100	0.999
Thickness of hemopericardium (mm)	14.7 ± 7.7	15.7 ± 7.9	12.9 ± 7.4	0.265
Malperfusion^a^ (n, %)	5, 12.2	3, 11.5	2, 13.3	0.866
DeBakey type II (n, %)	16, 39.0	10, 38.5	6, 40.0	0.923
Intramural hematoma (n, %)	8, 19.5	5, 19.2	3, 20.0	0.952

^a^Limb ischemia in one; stroke in four.

AR, aortic regurgitation; CPR, cardiopulmonary resuscitation; eGFR, estimated glomerular filtration rate; ED, emergency department; ESRD, end-stage renal disease; OR, operating room; SBP, systolic blood pressure.

### Surgical information

Table 2 provides detailed information regarding surgical variables. The vascular access of cannulation, extent of aortic repair procedures, incidence of entry tear exclusion, and CPB-related parameters were not statistically different between the two groups. The time span of CPB was 261.3 ± 104.7 min and 311.5 ± 66.1 min in the E-SXP group and E-CPB group, respectively (*P* = 0.103). The technical success rates of patient’s hemodynamics being stabilized after underwent rescue procedures were 76.9% (20/26) and 100.0% (15/15) for the E-SXP and E-CPB groups, respectively. Six patients in the E-SXP group underwent a subsequent E-CPB due to continuous bleeding from the dissected AsAo. Three patients underwent E-SXP in the ED. A total of 24.4% of the patients required Kerlix packing due to coagulopathy with uncontrolled bleeding, and 7.3% underwent extracorporeal membrane oxygenation installation in the OR due to intraoperative myocardial failure.

**Table 2:** Surgical information according to patient group.

Parameters	Overall	E-SXP	E-CPB	*P* value
	n = 41	n = 26	n = 15	
Femoral arterial cannulation (n, %)	40, 97.6	25, 96.2	15, 100	0.442
Axillary arterial cannulation (n, %)	19, 46.3	13, 50.0	6, 40.0	0.536
Aortic repair procedures				
Entry tear exclusion (n, %)	33, 80.5	20, 76.9	13, 86.7	0.448
Root replacement (n, %)	4, 9.8	3, 11.5	1, 6.7	0.613
Isolated AsAo replacement (n, %)	30, 73.2	17, 65.4	13, 86.7	0.138
Arch replacement (n, %)	7, 17.1	6, 23.1	1, 6.7	0.179
Partial arch (n, %)	5, 12.2	4, 15.4	1, 6.7	0.411
Total arch (n, %)	2, 4.9	2, 7.7	0, 0	0.271
Frozen elephant trunk (n, %)	1, 2.4	1, 3.8	0, 0	0.442
Cardiopulmonary bypass time (min)	279.7 ± 94.8	261.3 ± 104.7	311.5 ± 66.1	0.103
Aortic clamping time (min)	176.2 ± 64.8	184.7 ± 73.6	161.5 ± 44.5	0.276
Circulatory arrest time (min)	48.2 ± 24.0	45.9 ± 24.4	52.1 ± 23.4	0.428
ACP (n, %)	20, 48.8	14, 53.8	6, 40.0	0.393
RCP (n, %)	21, 51.2	12, 46.2	9, 60.0	0.393
Hypothermia temperature (°C)	20.0 ± 2.2	20.2 ± 2.1	19.7 ± 2.3	0.558
Delayed sternum closure^a^ (n, %)	10, 24.4	6, 23.1	4, 26.7	0.797
ECMO support (n, %)	3, 7.3	2, 7.7	1, 6.7	0.903

^a^Kerlix packing for uncontrolled coagulopathy and planned secondary exploration.

ACP, antegrade cerebral perfusion; AsAo, ascending aorta; ECMO, extracorporeal membrane oxygenation; RCP, retrograde cerebral perfusion.

### Postoperative complications

Similar high in-hospital mortality rates were observed in the E-SXP and E-CPB groups (30.8% versus 33.3%; *P* = 0.865) (Table 3). The blood transfusion volumes were also generally similar in the two groups. However, the E-CPB group showed prolonged ventilator dependence, intensive care unit (ICU) stays, and overall hospital course. The incidence of brain stroke was 15.4% and 33.3% in the E-SXP group and E-CPB group, respectively (*P* = 0.181).

**Table 3:** Postoperative mortality and morbidity according to patient group.

Parameters	Overall	E-SXP	E-CPB	*P* value
	n = 41	n = 26	n = 15	
Hospital mortality^a^ (n, %)	13, 31.7	8, 30.8	5, 33.3	0.865
Bleeding (n, %)	3, 7.3	3, 11.5	0, 0	0.172
Myocardial failure (n, %)	5, 12.2	3, 11.5	2, 13.3	0.866
Brain stem failure (n, %)	3, 7.3	0, 0	3, 20.0	0.018
Sepsis (n, %)	2, 4.9	2, 7.7	0, 0	0.271
Renal failure (n, %)	4, 9.8	3, 11.5	1, 6.7	0.613
Transfusion at 24 hr after surgery				
RBC^b^ (units)	9.8 ± 7.5	10.2 ± 7.8	9.1 ± 7.2	0.659
Plasma^c^ (units)	8.3 ± 5.1	8.5 ± 4.9	7.9 ± 5.6	0.688
Platelet (units)	17.6 ± 10.0	18.1 ± 10.2	16.8 ± 9.9	0.699
Re-operation for bleeding (n, %)	6, 14.6	2, 7.7	4, 26.7	0.098
Atrial fibrillation (n, %)	4, 9.8	1, 3.8	3, 20.0	0.093
Brain stroke (n, %)	9, 22.0	4, 15.4	5, 33.3	0.181
Infarction (n, %)	7, 17.1	3, 11.5	4, 26.7	0.215
Hemorrhage (n, %)	2, 4.9	1, 3.8	1, 6.7	0.686
Delirium (n, %)	11, 26.8	8, 30.8	3, 20.0	0.453
Seizure (n, %)	3, 7.3	3, 11.5	0, 0	0.172
Visceral ischemia (n, %)	3, 7.3	1, 3.8	2, 13.3	0.261
Limb ischemia (n, %)	1, 2.4	1, 3.8	0, 0	0.442
Malperfusion-related complication^d^ (n, %)	14, 34.1	8, 30.8	6, 40.0	0.548
Pneumonia (n, %)	9, 22.0	5, 19.2	4, 26.7	0.580
Extubation time (hr)	160.7 ± 251.9	96.7 ± 167.9	271.7 ± 331.9	0.030
Ventilator support >72 hr (n, %)	20, 48.8	9, 34.6	11, 73.3	0.017
Tracheostomy (n, %)	5, 12.2	3, 11.5	2, 13.3	0.866
ICU stay (days)	10.7 ± 15.4	5.5 ± 6.9	19.6 ± 21.4	0.025
ICU readmission (n, %)	0, 0	0, 0	0, 0	0.999
Hospital stay (days)	21.9 ± 18.4	16.7 ± 16.1	30.9 ± 19.3	0.015

^a^Death occurring during hospitalization.

^b^Red blood cell transfusion including amount of whole blood and packed red cell concentrate.

^c^Plasma transfusion including amount of fresh-frozen plasma and cryoprecipitate.

^d^Occurrence of postoperative renal failure, brain infarction, visceral ischemia, and limb ischemia.

ICU, intensive care unit.

### Regression analysis of in-hospital mortality

Table 4 shows regression analysis results among patients who underwent surgical rescues for ATAAD-complicated critical hemopericardium, including retrograde cerebral perfusion (RCP) during circulatory arrest and preoperative hemodynamic collapse requiring CPR. One significant prognostic factor for in-hospital mortality was identified: CPR (odds ratio 5.67; 95% confidence interval 1.08–29.65, *P* = 0.040).

**Table 4:** Logistic regression results for hospital mortality of patients who underwent surgical rescues for critical hemopericardium.

Parameters	β-coefficient	Standard error	Odds ratio, 95% CI	*P* value
Univariate logistic regression				
RCP	2.293	0.864	9.90 (1.82–53.83)	0.008
CPR	2.262	0.785	9.60 (2.06–44.74)	0.004
Multivariate logistic regression				
RCP	1.794	0.919	6.01 (0.99–36.39)	0.051
CPR	1.734	0.844	5.67 (1.08–29.65)	0.040

CPR, cardiopulmonary resuscitation; RCP, retrograde cerebral perfusion.

### Cumulative 3-year survival

**Fig 3:**
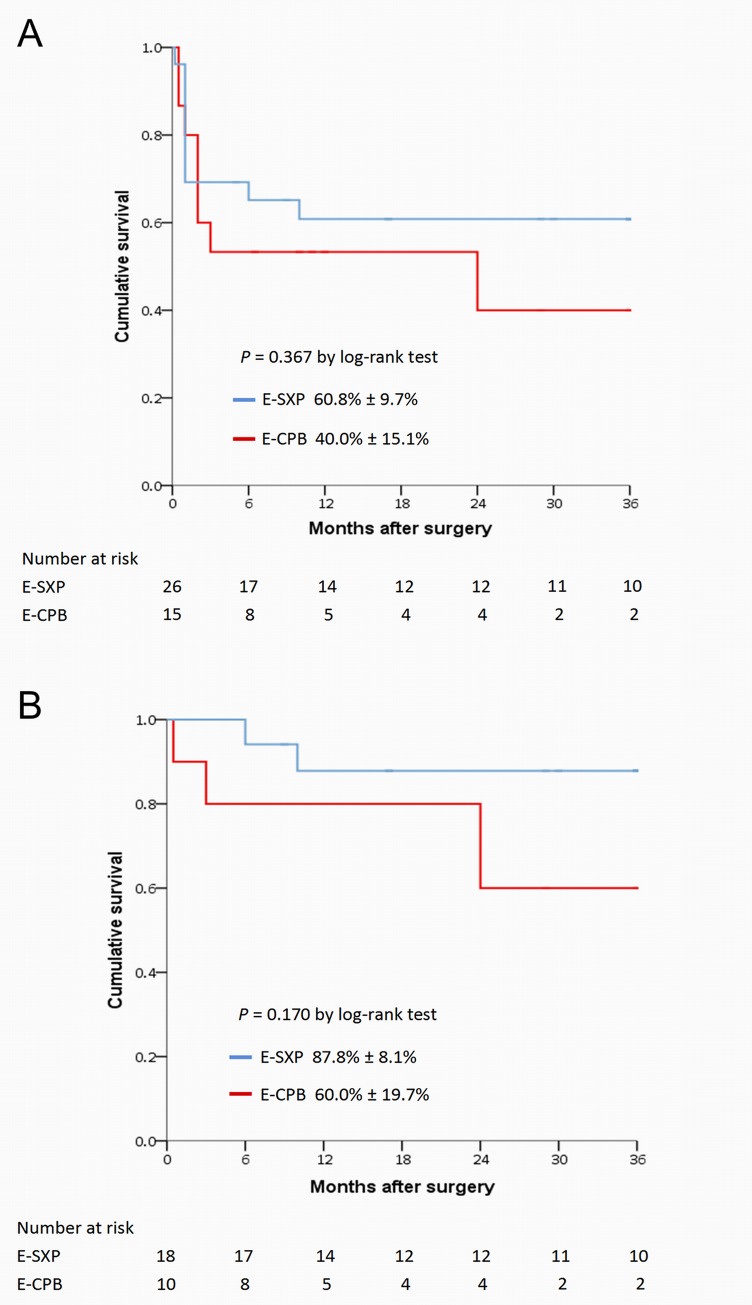
Three-year cumulative survival rates for 41 patients including in-hospital mortality (A) and for 28 patients excluding in-hospital mortality (B) stratified by rescue procedure.

As illustrated in Fig 3, the 3-year postoperative cumulative survival rates were not statistically different between the E-SXP group and E-CPB group, regardless of the inclusion of in-hospital mortality (60.8% ± 9.7% versus 40.0% ± 15.1%, *P* = 0.367; Fig 3A) or exclusion of in-hospital mortality (87.8% ± 8.1% versus 60.0% ± 19.7%, *P* = 0.170; Fig 3B).

## Discussion

ATAAD-complicated hemopericardium is common and dangerous due to increased risk of mortality and complications [2–4,10,11]. Once unstable hemodynamics are presented and refractory to medical treatment, emergent surgical rescues should be promptly implemented to bridge patients for further aortic repair procedures. However, the optimal strategy for managing this critical scenario remains controversial. In this single-institutional study of 41 patients who underwent surgical rescues for critical ATAAD-complicated hemopericardium, in-hospital mortality rates and postoperative complication rates were similar in the E-SXP group and E-CPB group. Furthermore, the mid-term outcomes also showed no inter-group disparity during a 3-year follow-up.

### Impact of hemopericardium

Hemopericardium and cardiac tamponade are leading causes of death in ATAAD [1,12], and these life-threatening conditions are commonly found in acute aortic syndromes, such as aortic dissection, intramural hematoma, penetrating atherosclerotic ulcer, and ruptured aortic aneurysm. These complications are characterized by rapid accumulation of hemorrhagic pericardial fluid under pressure which compresses all cardiac chambers, followed by compromise of systemic venous return to the right atrium and a limited cardiac output [13]. Furthermore, risks of organ malperfusion, neurologic deficits, and postoperative bleeding may also increase due to the influence of unstable hemodynamics and related consumption coagulopathy. According to previous studies [14,15], hospital mortality ranges from 43% to 54% and postoperative cerebrovascular complication rates are up to 33% in this high-risk subgroup. In the present study, we observed an inferior but acceptable outcome compared to that of our previous studies among the general ATAAD population [6,7]. High incidence of preoperative CPR and shock status may be important factors attributable for the poor outcomes. As illustrated in Table 4, preoperative CPR was an independent predictor of in-hospital mortality for ATAAD patients who underwent surgical rescues for critical hemopericardium. We suggest that in-time diagnostic studies, an aggressive strategy of surgical rescues and prompt management decisions are mandatory to quickly stabilize patients and optimize the outcomes in this high-risk population. Otherwise, most of these patients may not survive until surgery.

### Differences between E-SXP and E-CPB

According to the strategy of this institution, a routine invasive intervention is not necessary when hemodynamics do not suggest significant compromise and the amount of hemopericardium is small in volume. Without careful decision-making and accurate operation by the surgical team, the procedure itself may involve increased risk to patients. In the present study, approximately 80% of patients (150/191) with hemopericardium were successfully medically stabilized, and approximately 20% (41/191) were not. E-SXP and E-CPB were both effective surgical rescue procedures and provided similar postoperative outcomes. However, there may be some differences regarding the physiologic mechanisms, potential benefits and drawbacks between the two modalities.

As reported by Maisch et al., pericardiocentesis is a safe and effective procedure for treatment of cardiac tamponade caused by various underlying diseases [16]. However, in the context of hemopericardium-induced cardiac tamponade complicated by ATAAD, the indications for pericardiocentesis are still a matter of controversy, given the possibility that rapid and aggressive drainage of pericardial blood may precipitate a worsening leakage from the aorta into the pericardium [17]. Owing to the concerns about increasing instability of the dissected aorta and risk of rupture, the 2015 European Society of Cardiology guidelines for the diagnosis and management of pericardial diseases recommended pericardiocentesis in the setting of ATAAD with hemopericardium, with the recommendation that controlled pericardial drainage of very small amounts of the hemopericardium could be carried out to stabilize the patient temporarily in order to maintain blood pressure at approximately 90 mmHg [17,18]. However, this facility may not always be available in the OR, and an inexperienced echocardiography-guided pericardiocentesis may be less efficient and reliable compared to surgical rescue procedures. As a simplified technique, the E-SXP can decompress the pericardial cavity immediately and evacuate the hemopericardium. However, this procedure revealed similar potential risks of inducing large fluctuations in blood pressure and rupture of the dissected aorta. Even with a limited drainage with gauze packing at the subxiphoid wound, which mimics a controlled pericardiocentesis, there were still six patients who underwent concomitant E-CPB due to continuous bleeding from the aorta. Three of these patients died, including 1 died for sepsis with multi-organ failure on 27 days post operation, 1 died for massive bleeding on 1 day post operation, and 1 died for myocardial failure at 12 hours post operation. To prevent this disastrous outcome, we suggest that strict control of blood pressure/heart rate is mandatory when performing E-SXP in patients with ATAAD-complicated hemopericardium. Furthermore, E-SXP should be avoided for patients with signs of contained aortic rupture according to preoperative image studies, and a prepared CPB machine with blood recyclers should be on standby if possible.

In the present study, E-CPB was performed with a 100% success rate to temporarily stabilize hemodynamics. However, the E-CPB group showed a high incidence of brain stroke and hospital mortality induced by brain stem failure. We suggest several reasons for this finding. First, the E-CPB demands a more extended process, which is usually twice as long than that of E-SXP. The E-CPB procedure includes exposure of femoral vessels, cannulation, and connection of CPB circuits, which may be more complex than E-SXP. The interval of shock status, especially among patients with hemodynamic collapse requiring CPR, can induce considerable impacts on mortality and complication rates [19]. Furthermore, the malperfusion observed in ATAAD is highly correlated to complex anatomic interactions between the true lumen and false lumen along the entire dissected aorta. This anatomic interaction can be dynamic and even influenced by the surgical procedure itself. In the present study, all E-CPB procedures were performed via femoral cannulation, and a high incidence of RCP strategy was observed. We suspect the major reason for this finding is that surgeons preferred a more conservative strategy of exploring axillary artery after E-CPB in order to reduce the CPB time and its relative complications. In other words, E-CPB procedure was usually performed before patients were well prepared and sterilized. It usually took 20–30 min to complete these mandatory steps after E-CPB. Therefore, surgeons may try to minimize the prolonged CPB time if possible. In the institution, a double arterial cannulation with ACP strategy was commonly used in ATAAD patients with relatively stable hemodynamics [7]. However, this modality can not be a routine choice for managing this critical scenario in the present study. According to previous studies [20,21], cannulation with the femoral artery for ATAAD repair may induce more injury in the dissected aorta and end-organ malperfusion resulting from true vascular lumen compression or retrograde thromboembolization. As reported by Perreas et al. [22], RCP is associated with an increased risk of all types of neurologic complications and a trend toward increased 30-day and mid-term mortality compared to that with ACP in ascending aortic surgery. Therefore, patients who undergo E-CPB should be managed with a more aggressive strategy for detecting and treating neurologic complications early.

Prolonged ICU and hospital stays in the E-CPB group were observed in the present study. We suspect this inter-group disparity may be correlated with a bias regarding the etiology of hospital mortality. Bleeding and brain stem failure were the primary etiologies of hospital mortality in the E-SXP and E-CPB groups, respectively. In general, patients died for brain stem failure usually had a prolonged hospital course, and those who with massive bleeding may died in several hours post operation. The hospital stays of three patients died for bleeding in the E-SXP group and three patients died for brain stem failure in the E-CPB group were 0.3 ± 0.6 days and 39.7 ± 15.3 days, respectively.

In the present study, a trend of inferior 3-year survival was observed in the E-CPB group compared to the E-SXP group. We suspect this finding may by correlated with several factors, including extent of aortic repair procedures, bias from small sample size, and occurrence of cerebrovascular complication. During the 3-year following period, three patients of E-CPB group died and 2 of them had postoperative brain stroke; two patients of E-SXP died and none of them had postoperative brain stroke. The postoperative cerebrovascular complication may potentially affect the mid-term outcomes due to its relative neurological sequelae. However, an extended follow-up study should be conducted to clarify this issue.

**Fig 4:**
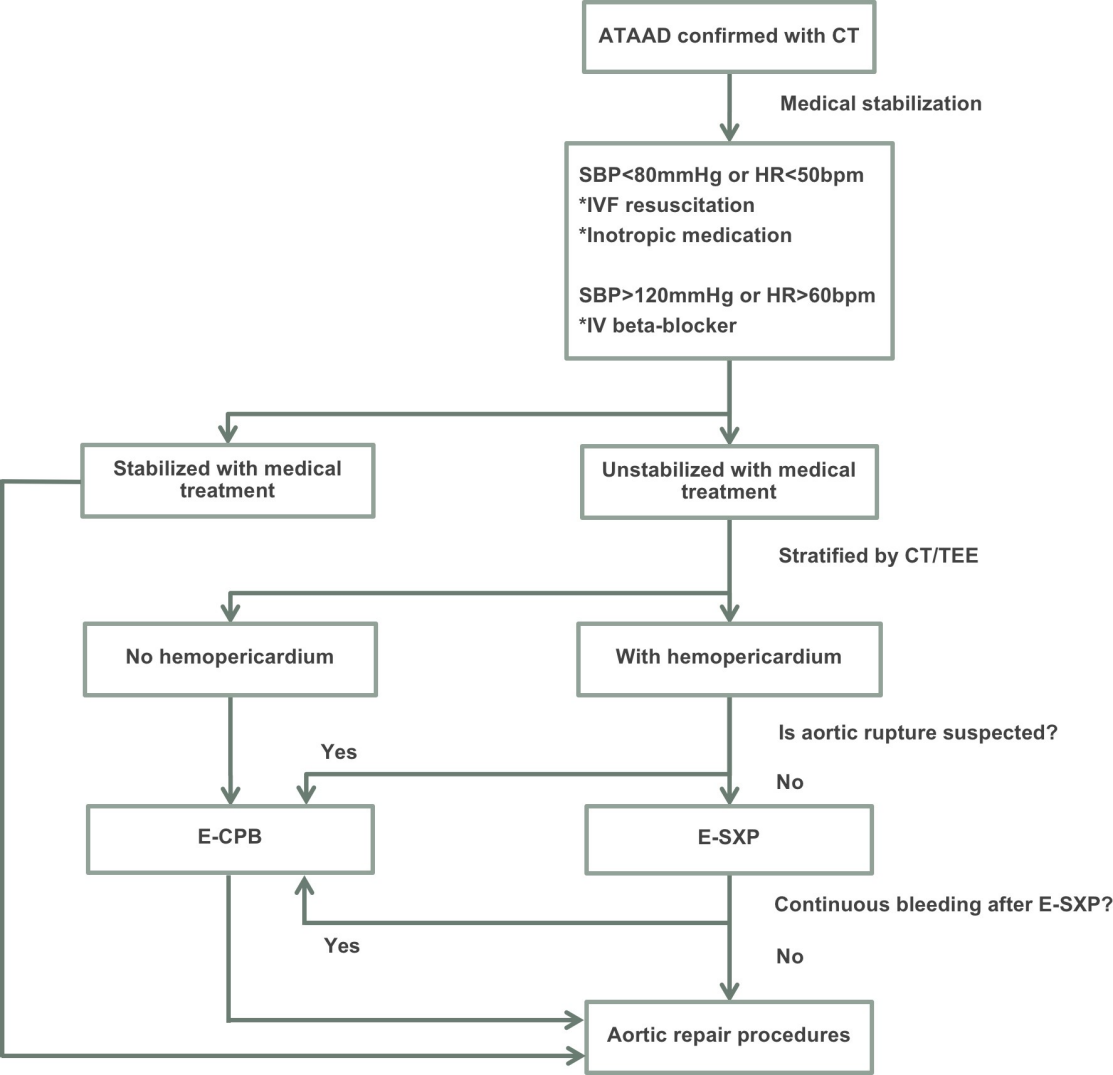
Flowchart of management for acute type A aortic dissection (ATAAD) patients with preoperative unstable hemodynamics and hemopericardium. CT, computed tomography; E-CPB, emergent cardiopulmonary bypass; E-SXP, emergent subxiphoid pericardiotomy; HR, heart rate; IV, intravenous; IVF, intravenous fluid; SBP, systolic blood pressure; TEE, transesophageal echocardiography.

According to the results in the present study, we propose a simplified therapeutic protocol for preoperatively medical stabilization and decision-making of surgical rescue for patients with unstable hemodynamics and ATAAD-complicated hemopericardium (Fig 4). The choice of rescue procedure was generally made according to clinical hemodynamics and image studies which evaluated the possibility of aortic rupture. If aortic rupture was not suspected, E-SXP was performed; if aortic rupture was suspected or persistent bleeding occurred after E-SXP, E-CPB was performed.

### Limitations

This study had several limitations. First, with a retrospective and non-randomized control design with small sample size, bias might exist that influenced the homogeneity and power of statistical analyses of the study groups. Second, the rescue procedure was chosen by individual physicians based on a clinical judgment according to patients’ preoperative information, and differences in surgeons’ experience for analyzing the image studies of insufficient quality, possibility of rapid disease progression, and concomitant factors of shock might potentially affect the strategy of surgical rescues. However, the bias regarding surgeons’ technical familiarity of rescue procedures was not found in the present study. Furthermore, some hemodynamic data, laboratory profiles, and inotropic medication dosage information were not completely analyzed due to incomplete records, which may have hindered more detailed analyses of physiological fluctuations during the perioperative course. Finally, despite the convincing mid-term results, an extended follow-up study should be conducted in the future to evaluate long-term outcomes in this high-risk population.

## Conclusions

Patients who undergo surgical rescues for critical hemopericardium complicated by ATAAD are a very high-risk population. The two rescue procedures showed similar short-term and mid-term outcomes. Careful decision-making regarding each individual patient and accurate management of the rescue procedure are crucial for good outcomes.
